# Optimization and control in bacterial Lag phase

**DOI:** 10.1186/1741-7007-11-120

**Published:** 2013-12-16

**Authors:** Daniel Schultz, Roy Kishony

**Affiliations:** 1 Harvard Medical School, Systems Biology Department, 200 Longwood Ave, Warren Alpert 519, Boston, MA 02115, USA

## Abstract

The lag phase of bacterial growth is important from a medical and food safety perspective, but difficult to study due to the low density and metabolic rate of cells. A new study by Alon and colleagues reveals that the gene expression program during early lag phase prioritizes carbon source utilization enzymes over genes responsible for biomass accumulation. This cellular strategy ultimately maximizes growth, making the best long-term use of the new nutrient-rich environment.

See research article: http://www.biomedcentral.com/1752-0509/7/136

## 

When cells in stationary phase are introduced into a rich environment, it is in their best interest to gain biomass as quickly as possible in an effort to secure resources before their competitors. What, then, is the role of the lag phase, an initial period when no growth is observed? Known about for over a century and still poorly understood, the lag phase has proven difficult to study due to the low density and low metabolic activity of bacteria during this phase. While it is usually interpreted as an adaptation period when the cell has to transition from low to high metabolism, little is known about the genetic program of the lag phase. A new study by Alon and colleagues [1] finds that gene expression during the lag phase was shaped by evolution to set the stage for maximal gain of biomass upon exit from lag phase by focusing on the production of bottleneck enzymes for carbon utilization.

Outside of the lab, bacteria spend relatively little time in exponential phase. Indeed, if given unlimited nutrients, a single *Escherichia coli* cell could grow exponentially into a colony the size of the planet in just a couple of days (exploited by Michael Crichton in his novel ’The Andromeda Strain’ [2]). Yet, modern studies have focused on the exponential phase alone, leaving the lag and stationary phases in obscurity. Understanding how bacteria behave when they are not dividing is of great importance for a number of applications. In food preservation, for instance, the maximum extension of shelf-life is directly related to the length of the lag phase [3]. In the case of bacterial infections, understanding the recovery from stationary phase is essential to explain the course of action of a pathogen once it reaches the bloodstream.

To further examine the lag phase, Alon and colleagues developed an automated assay that overcomes the limitations of having a low-density culture. The authors make use of a library of *E. coli* strains where each contains a different native promoter expressing a fluorescent reporter. Genes of interest were picked from this library, covering a variety of metabolic functions to allow the characterization of the lag phase expression program. For each gene analyzed, the wells of a 96-well plate containing fresh media were sequentially inoculated with the corresponding strain from the library at regular intervals, resulting in a time-series of the lag phase. The plate was then fixated on ice and analyzed in a flow cytometer for cell count, reporter fluorescence and cell size.

Two different stages of the lag phase are distinguished: an initial Lag1 where there is no production of biomass, and a later Lag2 where there is cell growth but no division. The duration of Lag1 depends on the new environment, and is most evident when cells are inoculated into poor media (media containing no amino acids and arabinose as a carbon source was used in this study). During this initial period there is no expression of ribosomal or amino acid biosynthesis genes. Instead, the resources are focused on the synthesis of carbon source utilization genes. Only when the newly synthesized transporters and enzymes guarantee a steady supply of carbon does synthesis of amino acids begin. This event lifts the stringent response, which diverts resources from growth into amino acid production, leading the cell into Lag2. At this stage, ribosomal and amino acid biosynthesis genes are finally expressed and the cell begins to grow in size, with gene expression already similar to the exponential phase. Accumulation of biomass starts slowly, and division times become increasingly faster in the first few generations (Figure 1).

**Figure 1:**
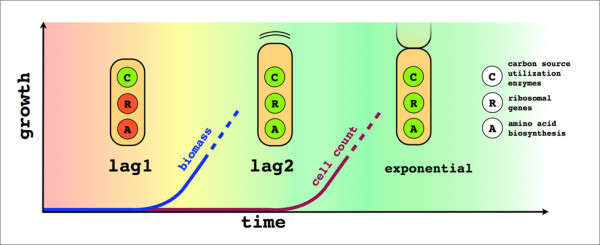
**The different stages of early bacterial growth upon inoculation into fresh media.** In Lag1 there is no accumulation of biomass, in Lag2 there is cell growth but no division, and in the exponential phase the population begins to grow. During Lag1 ribosomal genes and amino acid biosynthesis are shut down, preventing cell growth. The cell focuses its resources on the production of carbon source utilization enzymes, in a control strategy known as bang-bang, to maximize growth in the long run.

The fact that the cell does not begin accumulation of biomass as early as possible, and instead produces enzymes that will maximize the import and processing of primary resources to be used later on, addresses the question of how efficient evolution is in shaping gene regulation to optimize cellular processes. The processes responsible for gain of biomass are initially locked by the stringent response, while the genes responsible for carbon utilization are present in low levels, constituting a bottleneck for growth. What, then, is the optimal strategy of regulation to ensure the fastest accumulation of biomass in the long run? The authors used optimal control theory to analyze a cell growth model developed by Baranyi and Roberts [4], which includes bottleneck enzymes that make substrates available for biomass production. In agreement with the observed behavior, the optimal solution dictates that all resources should be initially invested in the production of the bottleneck enzymes alone, until a point in time where biomass production is switched on to full activation.

Control engineers have long known that such optimal control strategies where the control signal abruptly switches between on/off positions arise in a variety of minimum-time problems. Such strategies are known as bang-bang control. If one wishes to move an elevator stopped at one floor to a stop on another floor in the minimum time, the optimal control solution would be to apply maximum acceleration until a switching point where one should switch abruptly to apply maximum deceleration. Bang-bang control strategies seem to be applied to a variety of biological processes. Apart from being optimal in many situations, bang-bang control can also be easier to implement, since an on/off switch does not require complex regulation. In another study by van Oudenaarden and colleagues on the formation of mouse intestinal crypts, stem cells initially undergo only symmetric divisions, generating more stem cells until the entire adult pool is established. Only at this point the whole pool switches to asymmetric divisions, generating the crypt in minimal time [5].

How important is it to the cell that proteins are expressed at optimized levels? In a different study by Alon [6], the costs and benefits associated with the expression of the lactose utilization enzyme LacZ were determined, and optimal levels of expression were calculated for different concentrations of lactose. Strains grown under full induction of LacZ and fixed concentrations of lactose evolved to express the predicted optimal levels of the enzyme, highlighting the fitness advantage obtained by optimal expression [6]. In another study, they analyze the allocation of resources as the cell transitions from exponential to stationary phase - in other words, from a situation where growth genes are favored to one where stress responses are more important. The expression of relevant genes was found to follow Pareto fronts, which describe optimal allocation of resources, with the cell gaining just enough ability to fight stress at the expense of the minimal amount of growth, in an effort to optimize trade-offs [7].

However, cellular responses are not always optimized, and optimization for one condition may mean lack of optimization for another. When the cell is forced to deal with a temporary stress, otherwise optimized cell functions might operate inadequately. Cells exposed to DNA synthesis inhibitors, for instance, do not optimally regulate ribosomal genes, leading to an excess of ribosomes and an imbalance between DNA and protein synthesis rates [8]. Identifying and understanding a non-optimal cellular response can point to the Achilles’ heel of the cell, which may inspire novel antimicrobial approaches.

As for the understanding of the lag phase, many challenges still remain. While some cells try to maximize growth in the minimal time, others remain dormant for long periods, for reasons that are not completely clear. In fact, most microorganisms do not grow even when inoculated into yeast extract, which contains all the components of the metabolic network. Some species seem to wait for growth factors indicating the presence of other members of their complex societies, on whom they might depend [9]. Other species diversify their phenotypes between a majority of fast growers and a minority that remains in a low metabolic state, called persisters. This subpopulation is able to resist many types of stress and regenerate the entire population, and has been linked to latent bacterial infections [10]. As our knowledge advances, the initial part of the growth curve begins to show a wide complexity of behaviors and strategies, evolved to deal with the crucial mission of surviving in low numbers in a new environment.
